# Aligning Physical Literacy With Critical Positive Youth Development and Student-Centered Pedagogy: Implications for Today's Youth

**DOI:** 10.3389/fspor.2022.845827

**Published:** 2022-04-12

**Authors:** Fernando Santos, Tarkington J. Newman, Semra Aytur, Claudio Farias

**Affiliations:** ^1^Higher School of Education, Polytechnic Institute of Porto, Porto, Portugal; ^2^Higher School of Education, Polytechnic Institute of Viana do Castelo, Viana do Castelo, Portugal; ^3^Center for Research and Innovation in Education, Porto, Portugal; ^4^Department of Social Work, University of New Hampshire, Durham, NC, United States; ^5^Department of Kinesiology, University of New Hampshire, Durham, NC, United States; ^6^Department of Health Management and Policy, University of New Hampshire, Durham, NC, United States; ^7^Faculty of Sport, University of Porto, Porto, Portugal; ^8^Centre of Research, Education, Innovation, and Intervention in Sport (CIFI2D), Porto, Portugal

**Keywords:** pedagogy, learning, development, sport, physical education

## Abstract

The purpose of this article is three-fold: (1) revisit the concept of life skills to position physical literacy as a social justice life skill; (2) make the argument that physical literacy is particularly relevant within a critical positive youth development perspective; and (3) propose a novel critical praxis for developing physical literacy amongst youth. When considering emergent social issues, youth programming has the potential to integrate concepts from a range of theoretical frameworks, which may help youth transform into social change activists and competent movers. Such critical perspectives may guide sport and physical education programming as contemporary society poses numerous challenges concerning youths' diverse emotional, mental, physical, and social needs. In order to advance knowledge and practice, we urge researchers and practitioners to rethink the way sport and physical education has been traditionally conceptualized. Ultimately, we propose that educating individuals to recognize and then confront neoliberal values within a post-pandemic landscape is critical. These notions may help researchers (re)frame their positionality and readiness to tackle physical literacy through broader theoretical lenses which—as alluded to in this article—may equip practitioners and researchers to meaningfully advance social justice.

## Introduction

Within the United States Department of Health Human Services. (2018) reported that only 1 in 5 adolescents and 1 in 4 adults meet physical activity guidelines for aerobic and muscle-strengthening activities. Among children ages 6 to 12, less than 1 in 3 regularly participate in high-calorie-burning sports (e.g., basketball, biking, soccer, etc.) and less than 1 in 5 engage in no sport activities, trends that have held relatively steady between 2012 and 2018 (Aspen Institute, 2019). For children with disabilities, the rates are even more concerning as children with disabilities are 4.5 times less likely to engage in physical activity compared to children without disabilities. In the current landscape of COVID-19 pandemic, families have fewer options to engage in sports and other forms of organized physical activity. For instance, the Aspen Institute (2021) found that for more than 2 in 5 families, their community-based sports provider either closed, returned with limited capacity, or was forced to merge with another program. Making matters more complicated, 28% of children have lost interest in organized sports as of September 2021, up from 19% in June 2020 (Aspen Institute, 2021).

Further highlighting the need for a widespread and democratic development of physical literacy, health disparities and health inequities are known to disproportionally affect certain populations. For example, children from low-income homes face increasing barriers to participation, with children from the lowest-income homes being more than three times as likely to be physically inactive (Aspen Institute, 2019). Similarly, BIPOC (Black, Indigenous, and People of Color) youth and those living in poverty have lower initiation and duration of physical activity (Armstrong et al., 2018). In fact, Black children and youth have lost interest in sports more than any other racial/ethnic group since the beginning of the COVID-19 pandemic (Aspen Institute, 2021). Compounding this social injustice is that not only have BIPOC youth and those living in poverty been confronted with greater barriers to accessing health care (Agency for Healthcare Research and Quality, 2018) and health-promoting resources including greenspace (Locke et al., 2021). However, health outcome disparities (e.g., heart disease mortality among Blacks, diabetes mortality among Indigenous populations) have intensified over time (United States Institute of Medicine, 2012). Further, sport and physical education (and other forms of physical activity) have been considered spaces for social injustices, including lack of equality and episodes of discrimination (Love et al., 2019). To promote lifelong health, particularly among populations that have been historically marginalized, the inclusion of physical literacy should be deemed a priority for sport and physical education programming for youth (Telama and Yang, 2000; Tammelin, 2005).

With the objective of enhancing the development and transfer of physical literacy among all youth, the purpose of this article is to highlight the alignment between three novel youth-centric frameworks. Specifically, the person-based, development-oriented paradigm (PBDO; Kimiecik et al., 2020), *critical* positive youth development (CPYD) approach (Gonzalez et al., 2020), and adventure pedagogy (Newman et al., 2018) will be discussed as they relate to promoting the healthy development of youth and social justice promotion through physical education and sport.

Previous research has attempted to provide recommendations concerning how to enhance human health and development, especially among undeserved youth populations (Witt and Crompton, 1997; Lawson, 2005; Institute of Medicine, 2012). However, although aligned in purpose, these three novel frameworks have not yet been integrated. Independently, each framework offers innovative insights related to serving the diverse needs of youth in contemporary society. The need to place the focus on youth derives from the fact that young people face complex social challenges at a sensitive development period of their lives such as forging meaningful relationships with others, developing critical thinking as well as values and life skills (Lerner, 2021). Collectively, these youth-centric frameworks provide the theoretical groundwork for a *critical* praxis to promote physical literacy and the development of youth (Lewis et al., 2019).

To highlight the potential of this synergetic relationship, we build upon the work of Camiré et al. (2021) by *reimagining* traditional perspectives of PYD and conceptualizations of life skills. We argue that physical literacy is a social justice life skill, which positions youth to have an active role in building a more diverse, inclusive, and equitable society. This reimagined concept of physical literacy not only has the potential to contribute to one's holistic and healthy development but also empowers youth to foster socially just contexts to engage in sport, physical education, and other forms of physical activity (Farias et al., 2020). Drawing from PBDO and adventure pedagogy, we also contend that the development of physical literacy may help to promote the self-reflection, critical consciousness, and reflexivity necessary in order to actively confront the social injustices that are pervasive throughout society (e.g., health equity, health disparities, gender stereotyping, racism). Thus, physical literacy is postulated as a wide-reaching life skill that not only acts to promote development at the individual-level but also supports collective efficacy at the societal-level (Catalano et al., 2004; Cornish et al., 2020; Lerner, 2021).

### 21st Century Skills and Physical Literacy

From a holistic perspective and aligning with the requisite 21st century skills, physical literacy is recognized as a critical developmental concept when seeking to promote healthy youth development. Healthy youth development refers to the process through which individuals develop meaningful physical, emotional, social and cognitive skills that prepare them to face social challenges inherent to adult life (Cobo, 2013). Physical literacy is particularly relevant for youth who are in the midst of their maturation and are constantly learning, growing, and developing across developmental domains. The Society of Health and Physical Educators (SHAPE) America—which developed the National Health Education Standards—regards physical literacy as “the ability to move with competence and confidence in a wide variety of physical activities in multiple environments that benefit the healthy development of the whole person” (Mandigo et al., 2012, p. 28). Similarly, the Aspen Institute (2015) refers to physical literacy as “the ability, confidence, and desire to be physically active for life” (p. 9). Thus, physical literacy goes beyond motor competence and is intertwined with biopsychosocial development and transformative social learning, which accounts for all developmental domains (Jagers et al., 2019; Liu and Chen, 2021). Physical literacy refers to the development of behavioral, psychological, and physical components (Edwards et al., 2017).

The investment in promoting the healthy development of youth, specifically related to physical literacy, is at the core of many health service professions. For instance, the American Academy of Social Work and Social Welfare initiated the Grand Challenges for Social Work (2021), including the aims to *Ensure Healthy Development for Youth* and *Close the Health Gap*. Similarly, the United States Department of Health and Human Services and the Office of Disease Prevention and Health Promotion have identified health behaviors related to *Child and Adolescent Development* and *Physical Activity* as key objectives for their *Healthy People 2030* initiative. Furthermore, the United Nations General Assembly adopted Resolution 70/1—*Transforming our world: the 2030 Agenda for Sustainable Development*—which stated:

*We recognize the growing contribution of sport to the realization of development and peace in its promotion of tolerance and respect and the contributions it makes to the empowerment of women and of young people, individuals and communities as well as to health, education and social inclusion objectives (p. 1)*.

Ultimately, the United Nations Educational, Scientific, and Cultural Organization's (United Nations Educational, Scientific, and Cultural Organization, 2014) Quality Guidelines for Policymakers asserted that a nation's sustainable development relies heavily on building a socially just, equitable and safe world, comprised of healthy and well-educated youth. In this sense, physical literacy has been placed center-stage as an educational milestone that contributes to the full realization of human development.

Any educational practitioner (e.g., teacher, youth worker, coach) who aims to foster physical literacy through participation in sport and physical education (and/or other forms of physical activity) should ask themselves the following questions: What type of person do I want to develop through sport participation? What knowledge and skills should be developed in youth? How can I promote the development of youth to their fullest unique potential through their participation in sport? How can I empower youth to celebrate diverse perspectives and forms of participation that are inclusive of gender, race, language, culture, persons with disabilities, and re-embodiment?

The questions outlined above reflect 21st century elements that are needed to foster the learning and human development necessary for an innovative society (Cobo, 2013; Camiré et al., 2020). Such 21st century skills illustrate the importance of developing a multi-skills profile that includes capacities such as transdisciplinary knowledge, lifelong learning development, continual improvement of new literacies, as well as translation and adaptability. Underlying the notion of a multi-skills profile, self-direction, critical thinking, adaptability, creativity and innovation, as well as entrepreneurship also represent critical skills for youth to develop (Dede, 2010).

### Physical Literacy as a Social Justice Life Skill

Since the turn of the century, PYD frameworks, models, and approaches have been relied upon to promote the healthy development of youth (Lerner, 2021). Derived from ecological and developmental systems theories (Catalano et al., 2004; Camiré, 2021; Whitley et al., 2021), which asserts that dynamic interactions between social systems influence one's holistic well-being and development, PYD is a strengths-based perspective that holds that all youth have the potential for healthy development. Although PYD-based initiatives have been implemented in a variety of settings (e.g., school, afterschool, and community programs), the use of sport and other forms of physical activity to promote PYD has grown remarkably in recent years (Holt, 2016; Qi et al., 2020). In fact, research studying sport-based PYD^1^ has been conducted in a range of disciplines, such as sport psychology (Bean et al., 2020a,b), physical education (Santos et al., 2019), sport management (Lower-Hoppe et al., 2020), coach education (Camiré et al., 2020), social work (Newman, 2020), and disability studies (Aytur et al., 2018). This keen focus on sport-based PYD demonstrates the vested interest in promoting the healthy development of youth.

Similar to the diverse perspectives, outcomes associated with sport-based PYD are just as vast. However, several researchers have critiqued the concept of PYD due to its lack of operational clarity, as well as for providing vague and narrow notions about youth development (Haudenhuyse et al., 2013). Further, so called sport-based PYD programs have, in some cases, contributed to the notion that sport automatically leads to the development of life skills (Coakley, 2011). Nonetheless, empirical research has supported the intentional use of sport-based PYD to promote an array of healthy developmental outcomes.

From a biopsychosocial perspective, physical health outcomes incorporate physical fitness (Anderson-Butcher et al., 2019) and subjective health (Super et al., 2018), while mental health outcomes include psychological well-being (Cronin et al., 2018) and resilience (Vella et al., 2021). Further, intrapersonal psychological outcomes consist of self-identity (Newman et al., 2021), character (Weiss et al., 2019), and values (Koh et al., 2017), and academic performance (McDavid et al., 2019). Socially, youth participation in sport-based PYD program has been found to foster new social relationships (McDonough et al., 2018), a sense of belonging (Martin et al., 2016), as well as decreases in delinquency (Spruit et al., 2018). These positive outcomes have been postulated as components of a holistic perspective of healthy youth development, which emphases the importance of the *whole* person.

However, amongst the many healthy developmental outcomes associated with sport-based PYD (Bruner et al., 2021), life skills are often considered the most transferable PYD outcome that can serve as a bridge between self-efficacy and collective efficacy to promote social justice. Specifically, life skills are broadly recognized as “internal personal assets, characteristics, and skills such as goal setting, emotional control, self-esteem, and hard work ethic that can be facilitated or developed in sport and transferred for use in non-sport settings” (Gould and Carson, 2008, p. 60). Relatedly, life skills transfer is defined as:

*The ongoing process by which an individual further develops or learns and internalises a personal asset (i.e., psychosocial skill, knowledge, disposition, identity construction, or transformation) in sport and then experiences personal change through the application of the asset in one or more life domains beyond the context where it was originally learned* (Pierce et al., 2017, p. 194).

Although life skills can be conceptualized in a myriad of ways, they are traditionally thought of as psychosocial or socioemotional abilities (Winn et al., In Press). Further, in some cases, life skills are narrowed to skills that serve the purpose of helping individuals adjust to the status quo, adhering to a neoliberal mindset, and striving to meet society's success patterns (Camiré et al., 2021). However, as Camiré et al. (2021) proposed “there is a need to explore how PYD and life skills can be *reimagined* in order for youth sport research to be better positioned to promote dialogue and action on social justice issues” (p. 1). Thus, life skills that are thought of as competencies that guide individuals' efforts to promote a more socially just society have been positioned as *social justice life skills* (Quiroz-Martinez et al., 2005; National Healthcare Quality Disparities Report, 2018; Love et al., 2019). One example of a *reimagined* life skill (i.e., social justice life skill), which seeks to promote holistic development and reflects social (in)justice realities, is physical literacy. If physical literacy is developed, youth can be physically active individuals who have developed an array of social justice life skills that enable them to care for the well-being of others, overcome social challenges and systemic barriers, and promote physical activity (Petry and Jong, 2022).

A proposal to “re-dimension” the notion of life skills by coalescing with the construct of physical literacy argues that process-oriented developmental approaches can, for example, empower the explicit development of socially-oriented instruction decision making and youth ability to design meaningful developmental experiences. Youth can be endowed with the ability to autonomously build more democratic and inclusive contexts of participation in sport, physical education, and/or physical activity that build on and embrace the unique skills (strengths-based) of all youth with different levels of physical fitness and social background (Farias et al., 2020). Physical literacy, then, may be conceived as the embodiment of a disposition and building of a toolkit of life skills that are necessary to an individual's adoption of a healthful lifestyle as an integral part of their daily living (Whitehead, 2010; Edwards et al., 2017). The properties of physical literacy include those directed toward the growth of competent movers by emphasizing the development of the whole child with an emphasis on maximizing individual attributes, in a process achievable for all, regardless of initial skill set, sex/gender, age, fitness level, (dis)ability, and/or social background.

### Positioning a Novel Critical Praxis for Advancing Physical Literacy

Although sport and physical education have been increasingly used to foster physical literacy, traditional approaches have received criticism because “it is taken for granted in policy that physical literacy goes beyond the physical” (Quennerstedt et al., 2021, p. 11). Thus, we content that as a social justice life skill, physical literacy in contemporary society should be operationalized to reflect one's ability to engage in a range of physical activities in *multiple life domains* and *throughout the lifespan*, thereby promoting *holistic* development and spreading such notions across communities.

Aligning with Camiré et al. (2021), we posit that traditional concepts, including *youth development, positive youth development*, and *life skills*, must be challenged. A novel pedagogical praxis that appropriately situates physical literacy priorities within the demands of contemporary society may be needed to fully consider that life skills are indivisible from holistic human development. Further, there is a need for pedagogical approaches, programs, and policies that are designed to help youth develop into critically reflexive and active contributors in today's society (Ho et al., 2015).

For example, Outward Bound Adventures (OBA), which is the oldest non-profit in the nation, focuses on providing outdoor education, conservation, and environmental learning expeditions (Quiroz-Martinez et al., 2005; Honeycutt et al., 2015). This program is designed primarily for low-income, urban and rural youth and their families who would not otherwise have the opportunity to experience wild places and open spaces. OBA trips encourage long-term participation among BIPOC youth in environmental careers, civic engagement, and ecological and conservation activities. For instance, each year, approximately 800–1,000 youth and their families participate in OBA activities ranging from one-day trips to parks, forests, and monuments to 20-day expeditions.

To provide meaningful outdoor experiences and to foster partnerships that support economic opportunity and employment through community relationships, organizations like OBA have developed partnerships with the National Park Service, companies including Patagonia, and organizations such as the Outdoor Behavioral Healthcare Center. Other examples include the Better Family Life program (Ahmed, 2021) and the Sandhills Family Heritage Center (Aytur and Jenkins, 2009; Sandhills Family Heritage Center, 2020). Both organizations are community development corporations that embrace the *Sankofa* approach to healing in order to generate intergenerational cultural and economic growth within Black communities. The concept of “Sankofa” is derived from the Akan people of West Africa. “Sankofa” teaches us that we must go back to our roots in order to progress as a community and/or society, recognizing what our cultural roots have to teach us, so that we can achieve our full potential as we move forward. A third example is the Openlands program in Chicago. Openlands works to create a healthier urban forest, which provides the region with improved environmental, economic, and social benefits to local communities (Idrovo and Ebeling, 2021).

Another example from an international policy perspective is the country of Jamaica, which developed the Jamaican Sport Policy (JSP) framework in 1994, and continued to evolve its policy landscape through a series of iterative modifications that drew lessons from other developed nations including the Canadian Sport Policy. This resulted in the institutionalization of clearer policy objectives and strategies for monitoring and evaluation that aligned with the National Development Plan (Vision 2030 Jamaica) to facilitate public-private partnerships supportive of advancing inclusive sport (Government of Jamaica, 1990, 2009). For example, a partnership with Special Olympics Jamaica resulted in 14 new programs serving over 4000 athletes with disabilities since 2005.

### Lessons From the Person-Based, Development-Oriented Paradigm

The person-based, development-oriented paradigm (PBDO) is a person-focused and growth-based paradigm, which is centered upon the idea of human developmental plasticity. Always beginning with the person—not the behavior—Kimiecik et al. (2020) contend that youth “have an inherent agency to be directed toward positive well-being (broadly defined), and that by developing and experiencing core life goods (e.g., eudaimonia, self-determination, self-expression) will develop and sustain healthy lifestyles” (p. 492). In this way, PBDO represents a paradigm shift away from an exclusively behavior-focused model to embody a more holistic approach that focuses on individual strengths (rather than deficits). Importantly, we must clarify, even though it is centered on the person and not on behavior, it is always indispensable to consider outcomes such as the display of physical literacy properties, which practitioners and young people should aspire. However, we posit that this should not follow a one-size-fits-all perspective. Instead, each individual should be encouraged to pursue, develop, and manifest personally suitable properties as made possible by their unique capacity and historical, cultural and social background (and potential constraints) and perspectives of the world (Farias et al., 2020).

With the focus on health-promoting behaviors (e.g., self-regulation, self-esteem), a “PBDO approach refers to the notion that each individual has a central inner force that produces broad patterns of behaviors and experiences” (Kimiecik et al., 2020, p. 493). For instance, youth who develop self-determination (i.e., autonomy, competence, relatedness) have a positive perception of self, which in turn supports their intrinsic motivation for and inherent tendencies toward adapting health-promoting behaviors. Relating to health-risk behaviors (e.g., unhealthy diet, physical inactivity), PBDO seeks to promote inner strengths, positive psychological functioning, and a general sense of well-being that can prevent health-compromising and/or promote health-enhancing behaviors. Therefore, the emphasis is on mitigating health-risk behaviors through personal growth and development.

From a practical standpoint, PBDO underscores the critical importance of adult leaders *partnering* and *collaborating* with youth. From an experiential education perspective, facilitators of PBDO programs should empower youth by providing opportunities for youth to make meaningful decisions, take on leadership roles, try new things, have fun, and experience what it feels like to be their authentic selves (Newman et al., 2017). Although there are limited examples of PBDO-based physical activity programs, initial evidence highlights the importance of supporting what Kimiecik et al. (2020) refer to as “core life goods” (e.g., eudaimonia, self-determination) as a method for youth to develop and sustain healthy lifestyles, specifically related to physical activity. For instance, Amorose et al. (Amorose et al., 2016) found that youth with autonomy support from their coach and parent(s) had relatively high levels of self-determination motivation for sport participation. Anderson-Butcher et al. (2020) further demonstrated that coach and parent autonomy support significantly predicted health and fitness intentions, as well as physical activity self-efficacy. Other researchers also emphasized the role of physical activity in fostering meaningful social interactions, specifically compassion for others (Bartolomeo and Papa, 2019; Majed et al., 2021). Ultimately, PBDO is based on the notion that if youth feel better about their lives in a holistic and general well-being sense, they will be physically literate and potentially more critical toward the status quo (Kimiecik et al., 2020).

### Lessons From Critical Positive Youth Development

Considering how youth sport and physical education have been conceptualized (Lynch and Soukup, 2016; Gould, 2019), CPYD (Gonzalez et al., 2020) may contribute to a debate on how sport and physical education can be strategically used to contribute to the holistic and healthy development of youth. Borrowing from Freire's (Freire, 1972) work on critical consciousness, CPYD is a strength-based approach that highlights the need to foster PYD outcomes that aim to help youth become able to critically reflect about the current cultural and social norms. Furthermore, the aim of CPYD is to enable youth to develop actions across communities that may lead to social change. In other words, CPYD values “critical perspectives to understanding youths' environments and their interactions within them. Such critical understanding includes examining the role of systems—whether marginalizing or consciousness-raising—on youth development” (Gonzalez et al., 2020, p. 31). Such an asset-based approach to youth development aims to prepare youth to make a difference in today's society and take ownership for political stances that shift the current systems of oppression (Heberle et al., 2020). Beyond PYD, CPYD takes on a social justice approach toward youth development.

As positioned by Gonzalez et al. (2020), the 5 C's (caring, competence, confidence, connection and character) combined with the sixth C, contribution, may generate a 7th C called “critical consciousness.” Complementing previous research (Lerner, 2021), youth contribute to their communities through the 5 C's critical, as well as reflection and political efficacy. The goal of CPYD is that after youth develop the 5 C's, they take on the roles of transforming communities and spreading CPYD messaging (i.e., contribution through critical action). Contribution through critical action gives emphasis to the role played by youth as active learners and agents of change, capable of inspiring others to pursue goals such as thriving for social justice and physical activity and sport promotion in underserved communities (Mac Intosh et al., 2020). Thus, we posit that the CPYD approach, through leveraging the 7th C, can simultaneously build capacity at the population level for collective efficacy and foster greater participation in public policy processes capable of generating systemic change (Hipp, 2016; Butel and Braun, 2019).

From a practical standpoint, CPYD may help teachers and coaches explicitly and directly target psychosocial development, as well as create a climate that supports the development of multiple skills across life domains. Concurrently, may those use embody a CPYD perspective may become critical about the current status quo and the key social challenges that characterize contemporary society. Thus, the purpose of this approach to development is to empower youth to engage in critical action in their sociopolitical landscape, which may include engaging in a range of social justice issues within youth sport related to mental and behavioral health, racial and socioeconomic inequities, and physical health disparities, among other aspects.

### Lessons From Adventure Pedagogy

Grounded in experiential learning theory, Newman et al. (2018) forwarded that adventure pedagogy is a participant-centered approach that uses deliberately sequenced and structured activities with periods of reflection that promote the development of intrapersonal and interpersonal skills. This conceptualization of adventure pedagogy derives from the disciplines of physical education—which focuses on adventure-based learning—and social work—which focuses on adventure-based groupwork. Adventure-based learning “de-emphasizes the competitive, win-at-all costs mentality typically associated with sport and de-emphasizes the psychomotor goals in physical education” (Sutherland and Stuhr, 2014, p. 490). On the other hand, often used as a form of therapeutic intervention, adventure-based groupwork is designed to “promote social skills by engaging clients in experiential activities…[that] provide immediate and observable consequences of behaviors, and rely on problem-solving, while incorporating unfamiliar environments and the use of physical trust” (Norton and Tucker, 2010, p. 26). Trust is also a key component in the development of collective efficacy at the community and societal level (Hipp, 2016).

In addition to fundamentally being youth-centric, when applied to youth sport, Newman et al. (2018) proposed that adventure pedagogy consists of nine key tenets: (1) the promotion of PYD outcomes, (2) physical and emotional safety, (3) intentionally designed activities, (4) sequencing of prescribed activities, (5) a novel learning experiences, (6) intentional facilitation, (7) challenging group experiences, (8) real and immediate consequences, and (9) debriefing to transfer learning. For instance, safety pertains not only to physically safe environments that are clean and free from hazardous risks, but also the emotional safety of youth (Eccles and Gootman, 2002). In fact, adventure pedagogy places a precedence on self-determination and holds that youth should be empowered to choose their own level of involvement and challenge within activities. Activities that incorporate adventure pedagogy are often intentionally designed and sequenced in a way that requires youth to make meaningful decisions that result in real and immediate consequences. Thus, youth learn through their own unique lived experiences, rather than relying on a banking model of education that exclusively focuses on the perspectives of the educator (Freire, 1972). This unique pedagogical approach relies on collaborative partnership of facilitation and debriefing, rather than using a top-down approach when working with youth (Newman et al., 2017).

Research has demonstrated the alignment between adventure pedagogy tenets and healthy developmental outcomes for youth. For example, among a sample of youth from a community-based mental health center, research by Tucker et al. (2013) revealed that youth involved in adventure-based programming reported significant decreases in problem severity (i.e., related to fighting, skipping classes, using drugs/alcohol) compared to youth involved in counseling without an adventure component. Orson et al. (2020) also found that youth involved in an adventure-based program developed social-emotional skills, such as resilience, by preserving through emotional obstacles related to physical and social challenges.

Within the context of a community sport-based PYD program that infused adventure tenets, research by Newman and Anderson-Butcher (2021) illustrated that when staff took the time to have youth-centric conversations (i.e., facilitated and debriefed activities), youth understand how to use life skills (e.g., self-control, effort) in sport and demonstrated the significance of transferring those same life skills outside of sport to other life domains. Further, Bean et al. (2020a,b), although they did not explicitly use adventure-based pedagogy as an overarching framework, found that regardless of the sport context (i.e., recreational or competitive sport programs), coaches who valued the holistic development of youth sought to support youths' efficacy and mattering by integrating feedback from youth and providing leadership opportunities for youth. Taken together, adventure pedagogy provides a youth-centric approach that seeks to create meaningful learning opportunities.

### Integrating Theory Toward a “Reimagined” Physical Literacy

Upon critically reflecting on the current realities of youth, we advocate for a perspective of maximizing healthy development through participation in sport, physical education, and other forms of physical activity that align with the unique strengths of PBDO, CPYD, and adventure pedagogy. Although presenting an exhaustive mapping of such a novel critical praxis may not be feasible, we outline several action-oriented guidelines toward the implementation of such an approach.

As a foundational premise of PBDO and adventure pedagogy, our proposed critical praxis significantly departures from the traditional autocratic and directive approaches used by educational practitioners (e.g., teachers, youth workers, coaches). Such traditional approaches are often framed as one-size-fits-all perspective whereby individuals are stripped from their right to be challenged as individuals with unique sets of capabilities (physical, cognitive, and social) and potentials. Instead, our proposed critical praxis advocates for learning contexts that feature developmentally appropriate activities that intentionally and actively support the autonomy of youth. Further, these settings should be designed and scaffolded to gradually shift the responsibility toward youths' active construction of their own physical literacy skills while they develop through their critical (social) consciousness (see CPYD for such foundation). This should include the predominance of environments where explicit opportunities are provided to youth as a way in which to develop self-direction and critical thinking, contextual learning and adaptability, creativity and innovation, and entrepreneurship networking and collaboration—including social justice life skills that reflect democratic sport participation. This is an important recommendation that derives from CPYD and connects with recent work by Camiré et al. (2021).

#### Active Participation

Research has shown that the development of metacognitive skills (i.e., learning how to learn) through intensified participation in experiential learning experiences within autonomy-supportive environments is a meaningful way in which to empower youth to act in a self-determined way to promote more social just environments (Farias et al., 2020). When embodying this approach, educators not only act in partnership with youth, but gradually engage youth in the leadership and decision-making processes related to the instructional process (e.g., goal identification, task selection, peer-to-peer instruction, self- and peer-assessment) and issues related to other life domains.

For example, the abundant participation in peer-teaching and collaborative reflective exercises facilitates the group bonding and co-construction of deep knowledge of the strengths and limitations of each youth participant in the group. Through peer-teaching and peer-coaching activities youth become more sensitive toward, and aware of, the strengths, weaknesses, and learning needs of peers which may create opportunities to minimize social inequalities and help youth develop into competent and caring adults. Activities that engage youth in reflective observation and enable a deeper understanding of sport and life, as a result, may develop a greater sense of autonomy, self-awareness, and self-esteem. Embodied feelings of acceptance and satisfaction for the person one is generates a greater predisposition in children to accept difference and celebrate it in ways which negate prejudice and stereotyping and at the same time respect individual cultural identity.

#### Critical Reflection and Inclusive Experiences

Formally integrating into the pacing and development of the lesson or coach session activities social-based reflexivity and group social-oriented discussions promote initiatives that challenge established boundaries and stereotypical views. Pupils are actively encouraged to appreciate and view positively differences in others, whether arising from race, gender, ability or disability. The goal is to prompt youth to reflect about the impact of individual action on others as a way to promote the construction of collective meanings and community belonging. Educators can channel the intra- and inter-dialogue toward the development of critical consciousness regarding the diverse realities of youth and social landscapes. Dialogue themes may address power and privilege, discrimination and inequity, as well as diversity and inclusion. Such internal reflections and external dialogues may help to break down social stereotypes, discrimination, and bullying through empathy-centering exercises (“put yourself in the other's shoes”)—or may help to build more inclusive and equitable spaces.

Educational practitioners can also use their expertise to promote either heterogeneous (asymmetrical) or homogeneous (symmetrical) social and knowledge exchanges and prompt discussions about social justice. Youth themselves can be prepared to be active agents of social transformation throughout their everyday life interactions with others. Educators can mediate youth intervention toward building positive, democratic, and equitable learning dynamics among youth. Importantly, youth can be invested in learning to socially mediate the interactions among peers with a focus on identifying and acting on discriminatory, bullying, and/or inequitable social interactions, as well as sedentary behavior.

#### Bridging Holistic Youth Development and the Sociopolitical Landscape

Safe settings where all youth feel that they have opportunity to contribute legitimately to a debate centered around physical literacy as a multidimensional phenomenon and the current sociopolitical landscape is critical. Educators should find ways to connect the lived experiences of youth in sport to the sociopolitical landscape in which they live in. Thus, the educator's pedagogical approach needs to go beyond the sport context and consider impacts on other life domains. In a coaching setting, for example, the active inclusion of the family unit in youths' sport domain is an unavoidable (and desirable) reality. Avenues for extending the inter-contextual transfer of social justice life skills, such as physical literacy, call for promotion of activities that establish links between the learning context and life domains outside sport. Without such connection to the sociopolitical landscape, teaching and learning may become less meaningful and transferrable to youth's future as adults.

## Conclusion

As outlined above, there are multiple ways that educational practitioners and other key stakeholders can use these reflections to transform youth into active and reflexive contributors to society. We urge more applications to be developed in the future across a range of educational contexts to increase our understanding about how such strategies may increase the quality of youth's experiences in sport, physical education, and elsewhere. Therefore, the present article attempted to use the PBDO (Whitley et al., 2021) paradigm, CPYD, adventure pedagogy as novel approaches to promote physical literacy among youth who are confronted with a variety of challenges posed by contemporary society. For instance, the PBDO paradigm—a person-focused, growth-based perspective—acknowledges youth have intrinsic agency toward positive well-being and posits that by experiencing healthy development, youth will develop and sustain healthy lifestyles (Kimiecik et al., 2020). Similarly, CPYD not only recognizes the value of youth voices and the capacity of young people, but forwards that youth have the ability to challenge inequity and transform societal structures that sustain oppression by engaging in critical reflection and action (Gonzalez et al., 2020). Such foundations for framing youth development have been derived from Freire's work (Freire, 1972), which has highlighted the role of reflection and civic engagement. Finally, adventure pedagogy is defined as a participant-centered approach consisting of intentionally designed and sequenced activities that can lead to opportunities for growth and development when debriefed and reflected upon (Newman et al., 2018).

It should be noted that our objective is to reflect about the extent to which this conceptual unity may provide important guidance for educational practitioners (e.g., teachers, youth workers, coaches) intervention efforts, as well as for multi-sectoral partnerships involving educators, public health professionals, policymakers, environmental justice advocates, community members, and other stakeholders. However, this article represents a first reflexive exercise that should be further developed so researchers can better the link between learning, development and applications made throughout the lifespan and across sectors. Therefore, in closing, we offer three final considerations: (1) There is the need to conceptualize how teaching and learning occurs, and how these processes need to be structured to induce meaningful learning; (2) Development needs to be brought to this discussion and considered as the process through which changes across biopsychosocial domains will occur. These domains should not be separated and positioned as exclusive or incompatible as individuals have holistic needs; (3) It is important to consider that learning and development are framed in a way that considers the changing nature of society and enables youth to make applications throughout the lifespan. Further, key learning experiences and developmental outcomes attained across learning contexts—such as organized youth sport and physical education—should be transferable to other life domains.

Researchers can also create partnerships with community-based organizations utilizing participatory action research (PAR) methods such as photovoice, participatory photo mapping, and storyboarding that enable youth to act as “resident researchers” who operationalize physical literacy within the contexts of their lived experience (Wang et al., 2004; Arcaya et al., 2018). In the end, these notions may help researchers *reimagine* their positioning and research stance as they attempt to advance physical literacy through broader theoretical lenses which, as alluded to in this article, could hold the necessary potential to induce meaningful change to advance social justice.

## Data Availability Statement

The original contributions presented in the study are included in the article/supplementary material, further inquiries can be directed to the corresponding author.

## Author Contributions

FS conceptualized the manuscript. FS, TN, SA, and CF contributed to the writing and editing. All authors contributed to the article and approved the submitted version.

## Funding

This work was supported by the National Funds through the FCT-Fundação para a Ciência e a Tecnologia, I.P., under the scope of the project UIDB/05198/2020 (Center for Research and Innovation in Education, in ED) and by the University of New Hampshire.

## Conflict of Interest

The authors declare that the research was conducted in the absence of any commercial or financial relationships that could be construed as a potential conflict of interest.

## Publisher's Note

All claims expressed in this article are solely those of the authors and do not necessarily represent those of their affiliated organizations, or those of the publisher, the editors and the reviewers. Any product that may be evaluated in this article, or claim that may be made by its manufacturer, is not guaranteed or endorsed by the publisher.

## Footnotes

^1^Please note that the term “sport-based PYD” is an encompassing and inclusive term used to reference sport, physical education, and other forms of physical activity.
